# Are serious games an alternative to traditional personality questionnaires? Initial analysis of a gamified assessment

**DOI:** 10.1371/journal.pone.0302429

**Published:** 2024-05-02

**Authors:** Pedro J. Ramos-Villagrasa, Elena Fernández-del-Río, Ramón Hermoso, Jorge Cebrián

**Affiliations:** 1 Department of Psychology and Sociology, Faculty of Labour and Social Sciences, University of Zaragoza, Zaragoza, Spain; 2 Department of Computer Science and Systems Engineering, Faculty of Labour and Social Sciences, University of Zaragoza, Zaragoza, Spain; 3 Department of History and Social Sciences Applied to Design, Aragón School of Design, Zaragoza, Spain; University of Macerata: Universita degli Studi di Macerata, ITALY

## Abstract

Personality questionnaires stand as crucial instruments in personnel selection but their limitations turn the interest towards alternatives like game-related assessments (GRAs). GRAs developed for goals other than fun are called serious games. Within them, gamified assessments are serious games that share similarities with traditional assessments (questionnaires, situational judgment tests, etc.) but they incorporate game elements like story, music, and game dynamics. This paper aims to contribute to the research on serious games as an alternative to traditional personality questionnaires by analyzing the characteristics of a gamified assessment called VASSIP. This gamified assessment, based on an existing measure of the Big Five personality traits, incorporates game elements such as storyfication, immersion, and non-evaluable gamified dynamics. The study performed included 98 university students (77.6% with job experience) as participants. They completed the original personality measure (BFI-2-S), the gamified evaluation of personality (VASSIP), a self-report measure of the main dimensions of job performance (task performance, contextual performance, and counterproductive work behaviors), and measures of applicant reactions to BFI-2-S and VASSIP. Results showed that the gamified assessment behaved similarly to the original personality measure in terms of reliability and participants’ scores, although the scores in Conscientiousness were substantially higher in VASSIP. Focusing on self-reports of the three dimensions of job performance, regression models showed that the gamified assessment could explain all of them. Regarding applicant reactions, the gamified assessment obtained higher scores in perceptions of comfort, predictive validity, and attractiveness, although the effect size was small except for the latter. Finally, all applicant reactions except for attractiveness were related to age and personality traits. In conclusion, gamified assessments have the potential to be an alternative to traditional personality questionnaires but VASSIP needs more research before its application in actual selection processes.

## Introduction

Personality is one of the most important predictors in personnel selection [1]. It is usually measured through questionnaires due to its relationship with work outcomes, its ability to provide information different from other assessment methods, and because it exhibits relatively small group differences [2]. However, it is also known that personality questionnaires have limitations, like their relatively unfavorable applicant reactions [3] and the risk of faking [4]. With the rise of digital personnel selection, technology may overcome these limitations [5]. For instance, the use of game-related assessments (GRAs) instead of questionnaires provides adequate psychometrics, more favorable applicant reactions, and reduction of the risk of faking [6]. However, the accumulative research on GRAs highlights that not all games are the same [7] and that differences between them impact their alleged strengths [8]. According to future avenues of digital personnel selection [5], we need to study criterion validity and applicant reactions to each type of GRA [8, 9]. The study reported here contributes to this call by analyzing the psychometric properties and applicant reactions to a personality questionnaire transformed into a gamified assessment (i.e., the GRA closest to traditional assessment) and comparing its functioning with the original instrument.

### GRAs: Types and implications

GRAs are a set of assessment methods built on gamification, that is, the incorporation of game elements into a non-gaming context [8]. GRAs can be based on traditional assessments, including specific game elements (challenges, game fiction, immersion, etc.), or be actual games. Only GRAs whose main goal differs from fun are called *serious games* [10].

**Fig 1:**
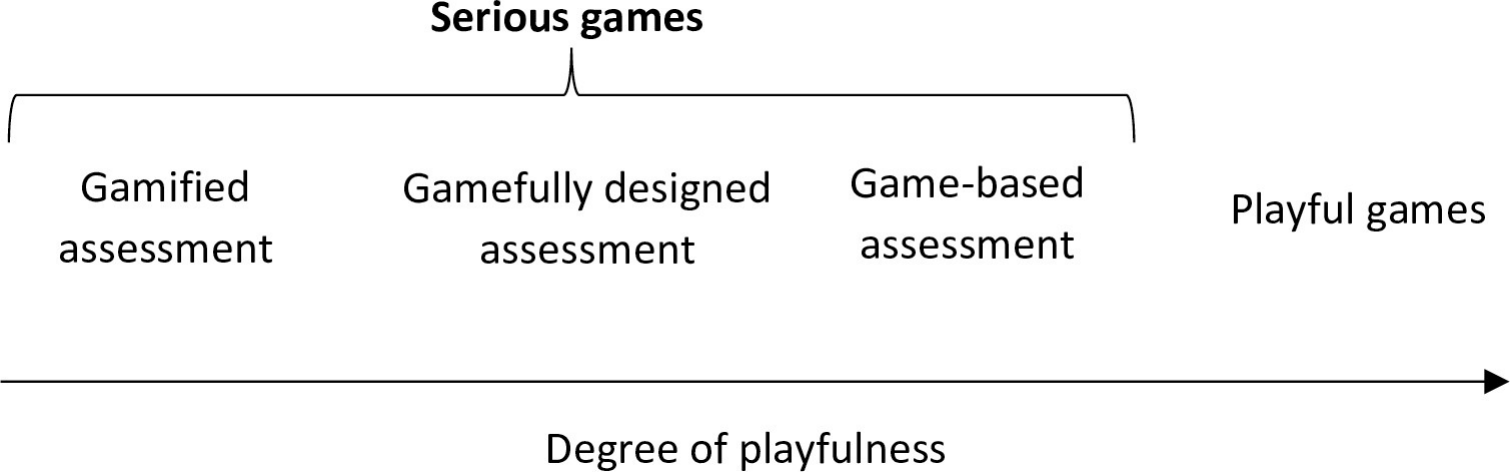
Types of GRAs.

Given their particularities, GRAs differ from well-established assessment methods like Likert-scale questionnaires, situational judgment tests, or simulations. However, they must still fulfill evaluation standards of reliability, validity, fairness, and bias [7]. As the investigation on GRAs progressed, it became clear that their design impacts their functioning, leading Ramos-Villagrasa and colleagues [8] to develop a taxonomy of GRAs. According to these authors, GRAs can be classified on a continuum based on their degree of playfulness, as depicted in Fig 1. At one end of the continuum are GRAs quite close to traditional assessments, and at the other end are playful games that were not designed for assessment, but are used for that purpose. The categories of GRAs are, from one end to another: gamified assessments, gamefully designed assessments, game-based assessments, and playful games. Below, we will describe each of these types.

Gamified assessments use psychological assessment know-how from the traditional approach and incorporate some elements from games to enhance the experience. As an example, they can be a Likert-scale response format embedded in the narrative (called *storyfication* [11]) or a situational judgment test with images and music to be more immersive [12]. Whatever gamification process is used, the assessment is still traditional, and the gamification process can probably be reversed (i.e., the gamified elements can be removed, and the assessment can still be performed). An example of gamified assessment is the gamified version of the Wisconsin Card Sorting Test [13].

Gamefully designed assessments are similar to gamified assessments but the gamification process is so embedded in how the assessment is performed that it cannot be undone. Thus, it may be considered a “hard” version of gamified assessment, as opposed to the aforementioned gamified assessments. An example of gamefully designed assessment is Owiwi [14], a situational-judgment test in which the item content can only be understood through the game’s narrative.

Game-based assessments are games that measure the constructs of interest from the user’s game behavior. The applicant’s score does not always correspond to the traditional way of assessment (e.g., answering questions or providing solutions with a limited amount of options). Instead, scores can be based on in-game behavior and/or user interaction with the game (reaction time, mouse clicks, etc.) An example of a game-based assessment is Nawaiam [9].

The last type of GRA contains games that are not serious games, but playful games. These games were developed for entertainment, but they are used for assessment under certain circumstances. An example is the virtual reality game *Job Simulator* [15].

According to the recent systematic review on GRAs in personnel selection [8], validity seems to be better in gamified assessments or gamefully designed assessments, whereas applicant reactions should be better in game-based assessments and playful games [8]. Nevertheless, this is only a proposition because the amount of research on each type of GRA is scarce. As the present study focuses on gamified assessments, we will delve into this type of GRA.

### What we know about gamified assessments

To understand the particularities of gamified assessments, we need to review the research conducted with them. To our knowledge, ten published studies have investigated gamified assessments. These studies use this type of GRA to measure: (i) personality [11, 12, 16, 17], (ii) cognitive ability [13, 18, 19], (iii) in-game performance [20, 21], and (iv) skills [22]. The content of the studies is related to validity, applicant reactions, and fairness. We will summarize their findings in the next paragraphs.

With regard to validity, the gamification process of gamified assessments does not seem to affect construct validity substantially [12, 16, 22]. Concerning criterion-related validity, there are no studies linking gamified assessments and job performance, and only one study used gamified assessments to measure cognitive flexibility as a predictor of academic performance [13]. This is an important issue because job performance is a capital variable in the work setting that refers to workers’ voluntary behaviors that impact organizational goals [23]. Within job performance, there are at least three main dimensions [24]: (1) task performance, which is related to in-role behaviors; (2) contextual performance, which includes behaviors that contribute positively to the organizational environment; and (3) counterproductive work behaviors, which comprises any deviant behavior directed at people or the organization and which are detrimental for organizational performance. If gamified assessments are considered for personnel selection, they must demonstrate their ability to predict criteria of interest to the organization, namely one or more dimensions of job performance. Other types of GRAs have been shown to have relationships with task performance or contextual performance [14, 25, 26].

Applicant reactions refer to individuals’ attitudes, affects, and cognitions concerning the personnel selection process [27]. They considerably impact applicant attitudes, intentions, and behaviors [3], such as accepting a job offer or recommending the employer to other applicants [28]. On the basis of affective events theory, attitude-behavior relations, and models of job performance, McCarthy and colleagues suggest considering applicant reactions as situationally-based or dispositionally-based [29]. Situationally-based applicant reactions are based on beliefs in the assessments and their development (e.g., fairness, job discrimination, test usefulness). On the other hand, dispositionally-based reactions are strongly based on personal characteristics like personality (e.g., test-taking anxiety, motivation, self-efficacy).

Addressing the call for further research on applicant reactions and technology [3], most research on gamified assessment is focused on reactions, demonstrating that the use of gamified assessments has a positive effect on many reactions [8]—both situationally-based and dispositionally-based. Regarding reactions related to the situation, gamified assessments are associated with high scores in consistency of administration/procedural justice [13, 19], ease of use [12], face validity [11, 13], fairness [11, 21], job relatedness/predictive validity [11, 19], opportunity to perform [19], organizational attractiveness [20], privacy and security [21], provision of selection information [19], and recommendation intentions [20]. Concerning reactions based on dispositions, affective reactions [13], effort [12], and enjoyment/satisfaction [12, 20, 21] are positive when gamified assessments are used. Some studies even compared traditional and gamified assessments, suggesting that reactions to GRAs are considerably more positive than those observed with conventional assessments [11–13]. The only reaction with mixed results is face validity: in a study with a composite measure of applicant reactions that include face validity, gamified assessment is considered to have higher face validity than the traditional approach [13] but, on the other hand, in another study that measures face validity individually, its scores are higher than in the original scale [11]. Nevertheless, the increase compared with traditional methods is not always sufficient to deserve substitution by a gamified assessment [22]. Moreover, being male, having high self-efficacy beliefs about technology, and perceived fairness may influence the impact of GRA on applicant reactions [19, 20].

Finally, concerning equity, one study shows that computer experience may positively impact GRA results but experience as a gamer does not [13]. There is also evidence supporting the idea that gamified assessment are resistant to adverse impact [16] and faking [11].

After reviewing the literature on gamified assessments, we found some gaps. First, there are differences within the same type of GRA, such as different implementations of game elements [30], which may also affect its functioning as an assessment method. Examples are gamified assessments measuring the Big Five that showed different ways of gamification: Landers and Collmus [11] and Hilliard and colleagues [16] using images instead of items, and Harman and Brown using illustrations to improve a storyfied assessment [12]. To describe which game design features impact the quality of measurement and applicant reactions, we need more primary studies with gamified assessments that use different strategies. Second, to support the idea that gamified assessments are useful for personnel selection and other Human Resources processes, such assessments should provide evidence of validity [9]. Considering prior studies, more evidence of criterion-related validity is needed, for example, to demonstrate that gamified assessment scores are associated with job performance and not only with academic performance. Third, given that prior literature suggests that gamified assessment promotes positive applicant reactions, the next issue is to know what personal variables (sociodemographic and psychological) enhance this effect.

### The present study: An analysis of VASSIP

Given the supposed advantages of GRAs and the fact that gamified assessments are the type of GRA closest to traditional instruments in personnel selection, it seems reasonable to view gamified assessments of personality as an alternative to traditional questionnaires. With this idea in mind, we aim to investigate the functioning of one of these tests: VASSIP.

VASSIP has been developed by the first three authors of the present paper based on an existing measure of the Big Five personality traits: the short version of BFI-2 [31]. The items and response format are the same as in the original scale (five-point Likert scale, ranging from *disagree strongly* to *agree strongly*). The assessment (“play a game”) can be performed in about 20–30 minutes. A description of the game following the taxonomy of game elements of Bedwell and colleagues [30] can be seen in Table 1.

**Table 1:** Description of VASSIP according to Bedwell and colleagues’ taxonomy.

Category	Description
1. Action language	The player must choose options displayed on the screen with the mouse.
2. Assessment	The player’s answers to each question score on one of the Big Five personality traits. The player does not have information about their scores throughout the game.
3. Conflict / Challenge	The story is linear, but some minor changes take place according to game decisions. The difficulty is low because any decision makes the story go on.
4. Control	The player chooses between different options at certain points of the story.
5. Environment	The story takes place in a space base in the far future.
6. Game fiction	The narrative and course of action have a high degree of realism.
7. Human interaction	There is no human interaction throughout the game.
8. Immersion	The game uses pictures, video, and music to enhance immersion.
9. Rules / Goals	The rules are clear and known to the player at the start of the game.

**Fig 2:**
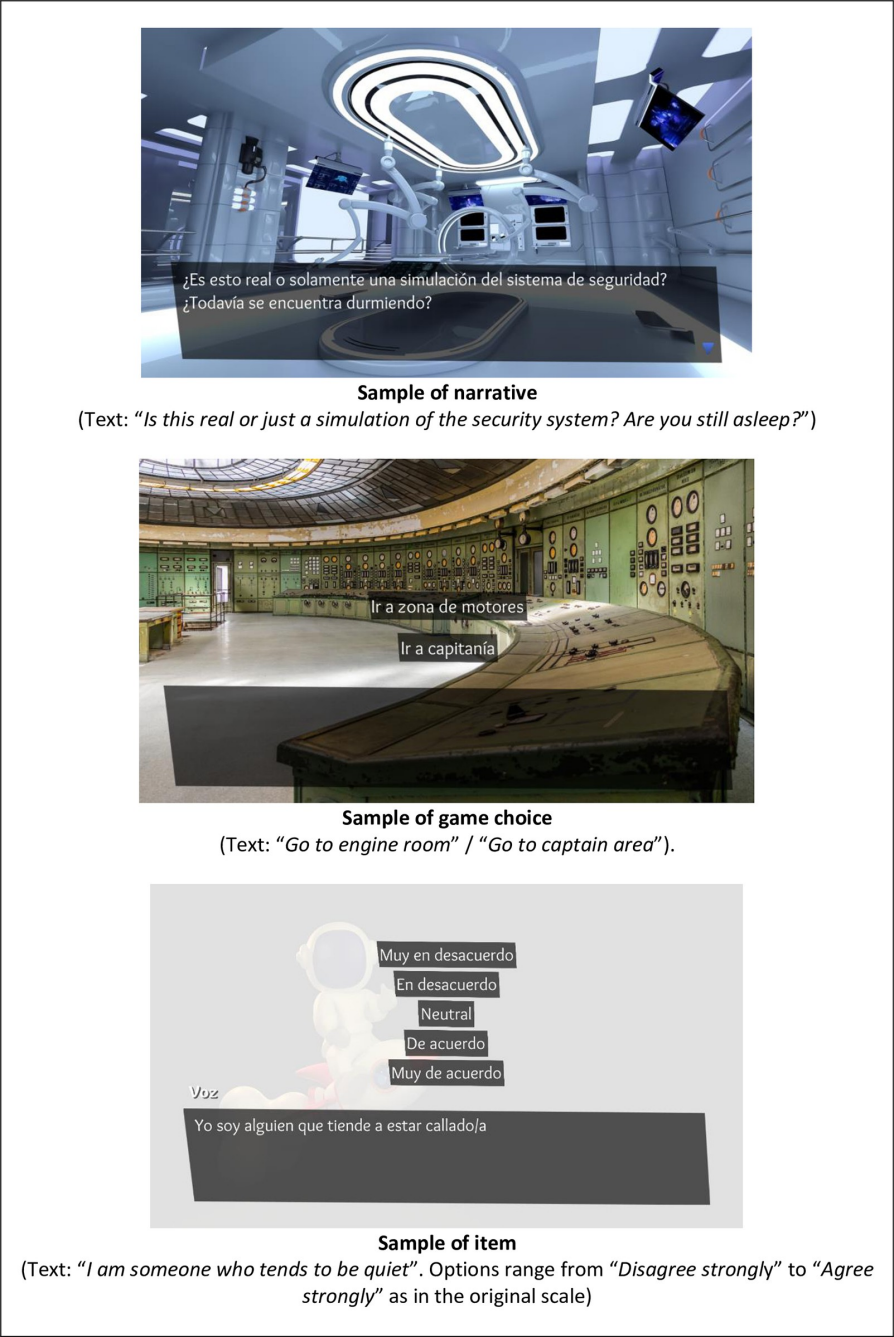
Screenshots of VASSIP.

The gamification process incorporated three game elements: storyfication, immersion, and non-evaluable gamified dynamics. Storyfication was done by incorporating a science fiction narrative. The applicant is part of the crew of a space base whose security has been compromised. Their role in the game is to overcome the security system, demonstrating to the artificial intelligence that the applicant is a human being, not an alien or an android. The story has three different endings (human, alien, or android) but the different endings do not impact the assessment process. In contrast to other gamified assessments based on storyfication [11, 12], the narrative was designed so that the participant responds to the same items as in the original test. Immersion in the game was developed by environmental music and images that illustrate the progress through the story. Some samples of VASSIP screenshots can be seen in Fig 2. Other gamified assessments that measure personality have used images to illustrate what happens in the story [12] or to replace items [16], but we consider that VASSIP offers a different experience by integrating images and music more immersively. The last game element is non-evaluable gamified dynamics. They were introduced by decisions and puzzles that the player must face throughout the game but that do not impact the applicant evaluation. Non-evaluable dynamics are used more often in GRAs that are closer to playful games, like gamefully designed assessments [9].

Having described VASSIP, we proceed to develop the hypotheses. In accordance with the gaps in the literature, we shall compare the original scale and the gamified version, investigating criterion-related validity, applicant reactions and the personal determinants of these reactions.

Regarding the comparison between the BFI-2-S and VASSIP, based on the aforementioned research [12, 16], we expect that the gamification process will not negatively affect construct validity because gamified assessments are very similar to traditional assessments. In other words, we expect participants’ scores to remain stable between assessment methods. Thus, our first hypothesis is as follows:

*H1*: *Personality traits measured with VASSIP will obtain the same scores as when these traits are measured with BFI-2-S*.

Continuing with validity, we want to investigate the criterion-related validity of VASSIP. Previous studies on gamified assessments do not provide evidence of associations with job performance. We want to determine whether VASSIP can do so. The relationship between the Big Five and job performance has been well-demonstrated in previous research with traditional assessments like questionnaires [32], suggesting that Conscientiousness and Negative Emotionality are somehow related to performance; Open-mindedness and Agreeableness are related to contextual performance, and the latter is also associated with counterproductive work behaviors [33–36]. Thus, we expect that VASSIP gamified assessment scores will obtain the same associations as the BFI-2-S.

*H2*: *Personality traits and job performance will display the same associations regardless of the assessment method (BFI-2-S or VASSIP)*.

Regarding applicant reactions, previous literature on GRAs is consistent about the fact that this kind of assessment is associated with positive applicant reactions [11–13, 19–21], and that these reactions are stronger than reactions to traditional assessments [11–13]. In our study, we focused on four reactions: comfort and predictive validity because they are considered relevant dimensions to examine the usefulness of a selection method [37]; fairness because it is capital to inclusive personnel selection [38]; and organizational attractiveness because prior research suggests that it shows the highest increase compared with the traditional approach [8]. Thus, we hypothesize:

*H3a*: *VASSIP will obtain higher applicant reactions than BFI-2-S*.
*H3b*: *Differences between VASSIP and BFI-2-S in perception of organizational attractiveness will display a higher effect size than the remaining reactions*.

Moreover, we also want to investigate the determinants of applicant reactions. Regarding sociodemographic characteristics, Ellison and colleagues [19] suggest that women are less prone to enjoy game-based content, but the effect size was small. Additionally, as young people have more experience with games and this experience is associated with positive reactions to GRAs [39], we expect age to display a negative association with applicant reactions. Regarding personality, investigation on its impact on reactions to gamified assessments is still pending but, based on studies on other methods, we can expect an association between the traits of Conscientiousness and Negative Emotionality and Fairness, and between Openness and all the applicant reactions due to the novelty of the method [40]. Therefore, we formulated our last hypothesis as follows:

*H4a*: *Being male*, *young*, *and obtaining high scores in Openness will be associated with more positive applicant reactions*.
*H4b*: *High scores in Conscientiousness and lower scores in Negative Emotionality will be associated with higher Fairness*.

## Method

### Participants

Participants were 98 Spanish university students (64.3% are women, *M*_age_ = 23.1 years with range 19–42; 59.2% are undergraduate students and the remaining are master-level students) who voluntarily agreed to be involved in the study. Most participants (77.6%) were working or had previous work experience. Of them, 29.5% worked in customer care and sales, 25.0% worked in intellectual professions, 11.4% in clerical positions, and the remaining in different posts. All participants received information about the research purposes and data usage before participating. Their informed consent was obtained in the first section of the online questionnaire that was applied to them (see Procedure section below). They also received a personalized report with their results in exchange for their contribution.

### Variables and instruments

#### Sociodemographic and work characteristics

We recorded gender, age, and job experience through an ad hoc survey.

#### Big Five personality–traditional assessment

We used the short form of the BFI-2 with the Spanish translation provided by the developers of the original questionnaire [31]. It comprises 60 items (12 per dimension) that measure Negative Emotionality (e.g., “[I am someone who…] is moody, has up and down mood swings,” observed α = .77), Extraversion (e.g., “is outgoing, sociable,” observed α = .61); Open-Mindedness (e.g., “is curious about many different things,” observed α = .66); Agreeableness (e.g., “is compassionate, has a soft heart,” observed α = .65); and Conscientiousness (e.g., “is systematic, likes to keep things in order,” observed α = .69) rated on a five-point Likert scale, ranging from 1(*disagree strongly*) to 5 (*agree strongly*).

#### Big Five personality–gamified evaluation (VASSIP)

We used the BFI-2 [31] adapted as described in the Introduction. Items, rating scale, and dimensions were the same as in the original measure. Observed reliability for the gamified evaluation was α = .85 for Negative Emotionality, α = .71 for Extraversion, α = .78 for Open-Mindedness, α = .69 for Agreeableness, and α = .67 for Conscientiousness.

#### Self-reported job performance

Task performance, contextual performance, and counterproductive work behaviors were measured using the Spanish version of the Individual Work Performance Questionnaire [23, 41]. This is a self-report instrument rated on a 5-point Likert scale, ranging from 0 (*seldom*) to 4 (*always*) for task performance and contextual performance, and from 0 (*never*) to 4 (*often*) for counterproductive work behaviors. Each dimension is composed of items that describe behaviors that may occur in any job [42]. Participants should answer thinking about their behaviors at work during the last three months. Dimension scores were computed by the mean values of each dimension. The number of items, observed reliability, and sample items were as follows: Task Performance (5 items, α = .77, “I knew how to set the right priorities”), Contextual Performance (8 items, α = .85, “I took on extra responsibilities”), and Counterproductive Work Behaviors (5 items, α = .77, “I complained about unimportant matters at work”).

#### Applicant’s reactions

Four types of applicants’ reactions were analyzed, all rated from 1 (*strongly disagree*) to 5 (*strongly agree*): perceptions of Fairness, Comfort, Predictive Validity, and Organizational Attractiveness. The higher the score, the more positive the applicant’s reaction. All of them are situationally-based reactions [29] because our study focused on GRAs as an assessment method. Fairness perception was measured with the 4-item scale by Kluger and Rothstein [43]. Observed reliability was α = .79 (traditional assessment)/.72 (gamified evaluation); a sample item is “I think this test/game is fair.”) Comfort and Predictive Validity perceptions were measured with the adaption of the Employment Interview Perceptions Scale (EIPS) [37] created by Ramos-Villagrasa and colleagues [9]. The scales can be seen in the Appendix. Comfort Perception was measured with 5 items (α = .65 for traditional assessment/.64 for gamified assessment; a sample item is “I have felt comfortable with the test questions/while playing the game”). Predictive Validity was measured with 6 items (α = .75 for traditional assessment/.80 for gamified assessment; a sample is “This test/game facilitates decision-making for recruiters.”). The last reaction considered was Organizational Attractiveness Perception, measured with the 5-item scale developed by Highhouse and colleagues [44]. As the participants were not involved in a selection process, they were asked to answer while thinking about an application for a hypothetical company that would use this test/game. Its observed reliability was α = .85 for traditional assessment/.90 for gamified assessment, and a sample item is “A job at this company is very appealing to me.”).

### Procedure

Participants were recruited from the research team’s students, offering them an opportunity to be evaluated "as in actual selection procedures" and receiving a personalized personality score report as a reward. They were involved in a 2-hour assessment session with three parts. In the first part, they were informed about the research procedure, the anonymous treatment of data, and their rights according to American Psychological Association (APA) standards. In the second part, they completed an online questionnaire with sociodemographic variables, personality measured by traditional assessment, job performance, and applicant reactions to traditional assessment. In the third part, they played VASSIP and completed an online questionnaire of applicant reactions to gamified assessment. They were allowed to rest for about 10 minutes between the two parts of the assessment. People who completed the assessment session received the personalized report by e-mail.

### Analyses

The analyses were descriptive statistics, Cronbach’s alpha, correlations, *t*-tests, and regression analyses. All analyses were performed with JAMOVI v.2.3. The database used in this study can be downloaded from Zenodo repository: https://doi.org/10.5281/zenodo.10500532.

## Results

Descriptive statistics (mean, standard deviation, skewness, kurtosis), displayed in Table 2, showed values that followed a normal distribution. The only exception was age, whose distribution was right-skewed and leptokurtic. This could be because the data were collected among university students, and most of them were around 20 years old.

**Table 2:** Descriptive statistics and mean differences between traditional assessment and gamified assessment.

Variables	*M*	*SD*	Skewness	Kurtosis	*t*	*p*	Cohen’s *d*
Gender	0.64	0.48	-0.606	-1.670			
Age	23.10	4.03	2.370	6.650			
Negative Emotionality	15.90 / 15.80	4.47 / 4.55	0.667 / 0.563	-0.118 / -0.597	0.518	.606	0.052
Extraversion	22.70 / 22.60	3.28 / 3.44	-0.272 / -0.352	-0.152 / -0.567	0.381	.704	0.038
Open-Mindedness	22.20 / 22.00	3.55 / 3.76	-0.358 / -0.300	0.526 / -0.359	0.942	.348	0.095
Agreeableness	24.40 / 24.60	3.40/ 3.12	-0.423 / -0.396	-0.594 / -0.176	-0.664	.508	-0.067
Conscientiousness	23.40 / 24.90	3.82 / 2.85	-0.391 / -0.262	-0.450 / -0.347	-0.657	< .001	-0.664
Task performance	2.99	0.58	-0.552	-0.178			
Contextual performance	2.61	0.84	-0.397	-0.085			
Counterproductive work behaviors	1.27	0.64	0.299	0.326			
Perception of Fairness	12.80 / 13.10	2.82 / 2.54	-0.912 / 0.026	1.410 / -0.060	-0.829	.409	-0.084
Perception of Comfort	18.90 / 19.90	2.72 / 2.38	-0.880 / -0.769	0.788 / 1.03	-4.482	< .001	-0.463
Perception of Predictive validity	18.60 / 18.90	3.75 / 3.86	-0.385 / -0.430	0.341 / 0.338	-1.130	.026	-0.114
Perception of Attractiveness	18.40 / 21.40	3.22 / 2.68	-1.000 / -0.632	1.670 / 0.790	-10.541	< .001	-1.064

*Note*. Gender: 0 = men; 1 = women; in columns with two values, the first one refers to BFI-2-S and the second one to VASSIP.

Our first hypothesis proposed that Big Five personality scores would be the same using BFI-2-S and VASSIP. According with Table 2, Conscientiousness mean score using VASSIP was substantially higher than using BFI-2-S, with an effect size of 0.663. Thus, we consider H1 as partially supported.

The second hypothesis proposes that VASSIP scores would be associated with job performance dimensions and in the same way as BFI-2-S. The associations between variables can be seen in Table 3. The relationships between personality traits and performance displayed the same pattern in the traditional assessment ($|\bar{r|}$ = .534) and gamified assessment ($\bar{|r}|$ = .493) except for Extraversion, which was associated with task performance only in traditional assessment (*r* = 243, *p* = .016), and the relationship between Conscientiousness and contextual performance, which was considerably higher in the gamified method (*r* = 287, *p* = .004 for traditional assessment and *r* = .375, *p* < .001 for gamified assessment).

**Table 3:** Correlations between variables.

Variables	1	2	3	4	5	6	7	8	9	10	11	12	13	14	15	16	18	19	20	21	23	24
1. Gender	—																					
2. Age	.064	—																				
3. Negative Emotionality_*tr*_	**.209**	.062	—																			
4. Extraversion_*tr*_	-.006	-.064	**-.202**	—																		
5. Open-Mindedness _*tr*_	.123	.016	-.050	**.286**	—																	
6. Agreeableness_*tr*_	.107	.049	-.106	**-.208**	-.071	—																
7. Conscientiousness_*tr*_	**.216**	.106	-.075	.198	.159	**.207**	—															
8. Negative Emotionality_*ga*_	**.226**	-.041	**.802**	**-.199**	.104	-.154	-.020	—														
9. Extraversion_*ga*_	-.076	-.039	-.175	**.805**	**.299**	-.163	.113	**-.245**	—													
10. Open-Mindedness _*ga*_	.126	.018	.044	**.286**	**.808**	-.057	.121	.039	**.335**	—												
11. Agreeableness_*ga*_	.136	.092	-.072	-.099	-.073	**.829**	.151	**-.212**	-.055	-.065	—											
12. Conscientiousness_*ga*_	**.244**	.148	-.005	**.333**	.072	.194	**.736**	-.119	**.266**	.140	**.211**	—										
13. Task performance	**.211**	.008	**-.214**	**.243**	.045	.149	**.524**	**-.222**	.142	-.002	.075	**.512**	—									
14. Contextual performance	.134	.076	-.182	**.437**	**.386**	-.093	**.287**	-.122	**.408**	**.345**	-.115	**.375**	**.411**	—								
15. Counterproductive work behaviors	.050	.051	**.322**	.058	.179	**-.262**	-.069	**.329**	-.019	.080	**-.273**	.042	-.064	.090	—							
16. Perception of Fairness_*tr*_	.161	-.088	-.036	-.085	-.098	**.255**	**.296**	-.129	-.116	.040	.170	**.246**	**.331**	-.012	**-.218**	—						
18. Perception of Comfort_*tr*_	.098	-.151	**-.026**	.186	.040	.123	**.206**	**-.276**	.073	.092	.101	.143	**.326**	.000	-.155	**.550**	—					
19. Perception of Predictive validity_*tr*_	**.257**	-.081	-.020	.040	-.045	**.274**	**.356**	-.030	-.066	.180	.147	**.324**	**.261**	.071	-.160	**.653**	**.441**	—				
20. Perception of Attractiveness_*tr*_	**.311**	-.117	-.016	-.021	-.127	.149	**.274**	-.013	-.126	-.057	.068	**.227**	**.226**	.066	-.169	**.396**	**.373**	**.486**	—			
21. Perception of Fairness_*ga*_	**.200**	-.051	-.169	-.102	-.063	.170	.137	-.140	-.043	.059	.032	.166	.198	.189	-.133	**.493**	**.261**	**.575**	**.306**	—		
23. Perception of Comfort_*ga*_	-.010	-.126	-**.264**	**.303**	.054	.118	.087	**-.279**	.191	.123	**.199**	**.224**	**.285**	.132	-.169	**.354**	**.576**	**.296**	**.260**	**.282**	—	
24. Perception of Predictive validity_*ga*_	**.260**	.033	-.158	-.064	.029	**.268**	.197	-.084	-.057	.171	.113	**.259**	.154	.128	-.121	**.339**	.196	**.661**	**.316**	**.714**	**-.275**	—
25. Perception of Attractiveness_*ga*_	.078	-.109	-.141	.049	-.004	.014	.160	-.071	.005	.115	-.023	.158	.070	.172	-.153	**.285**	**.327**	**.318**	**.515**	**.473**	**.494**	**.499**

*Note*. Bold values are significant associations (up to |.259| = .05; between |.260| and |.326| = .01; higher values = .001). Gender: 0 = men; 1 = women; *tr* = traditional assessment; *ga* = gamified assessment.

Focusing on VASSIP scores, task performance was associated with Conscientiousness (*r* = 512, *p* < .001) and Negatively Emotionality (*r* = -.222, *p* = .028), as was expected. With contextual performance, however, two of the expected personality traits did not display a significant association (Agreeableness: *r* = -.115, *p* = .258; and Negatively Emotionality: *r* = -.122, *p* = .230), whilst Extraversion did (*r* = .408, *p* < .001). Counterproductive work behaviors were associated with Agreeableness (*r* = -.273, *p* = .007) and Negatively Emotionality (*r* = .329, *p* < .001), but not with Conscientiousness (*r* = .042, *p* = .680).

Going deeper with the relationship between personality scores and job performance, we developed regression models with Big Five as predictors and job performance as criteria (task performance, contextual performance, and counterproductive behaviors). As we had two different measures of Big Five, we developed a model for the traditional assessment and another one for the gamified assessment. Results are displayed in Table 4. Regarding task performance, both assessments had the same predictors: Negative Emotionality (β = -.179, *p* = .041 / β = -.204, *p* = .029), and Conscientiousness (β = .470, *p* < .001 / β = .542, *p* < .001) with an explained variance of 33.2% and 32.0% respectively. Contextual performance had the same explained variance using both methods (30.3%) but with different determinants: Extraversion (β = .239, *p* = .020 / ns), Open-Mindedness (β = .289, *p* = .002 / β = .263, *p* = .006), and Conscientiousness (β = .223, *p* = .019 / β = .328, *p* < .001). It is true that Negative Emotionality was expected to be a predictor of contextual performance, but this was not the case even with the traditional approach. With regard to counterproductive work behaviors, the explained variance was higher for the traditional approach (29.7% vs. 24.9%) but with the same determinants: Negative Emotionality (β = .388, *p* < .001 / β = .373, *p* < .001) and Agreeableness (β = -.252, *p* = .009 / β = -.244, *p* = .012). According with all these results, we considered hypothesis 2 as partially supported.

**Table 4:** Hierarchical regression analyses.

**Traditional approach**	*Task performance* (*R*^2^ = .332)				*Contextual performance* (*R*^2^ = .303)				*Counterproductive work* *behaviors* (*R*^2^ = .297)			
*Predictors*	B	SE	β	*p*	B	SE	β	*p*	B	SE	β	*p*
Negative Emotionality	-0.023	.671	-.179	.041	-0.018	.017	-.093	.295	0.056	.013	.388	< .001
Extraversion	0.249	.011	.141	.156	0.062	.026	.239	.020	0.019	.020	.100	.325
Open-Mindedness	-0.009	.017	-.058	.523	0.069	.022	.289	.002	0.032	.017	.176	.060
Agreeableness	0.015	.015	.089	.338	-0.018	.023	-.074	.432	-0.048	.018	-.252	.009
Conscientiousness	0.071	.016	.470	< .001	0.049	.021	.223	.019	<0.001	.016	.003	.973
**Gamified assessment**	*Task performance* (*R*^2^ = .320)				*Contextual performance* (*R*^2^ = .303)				*Counterproductive work* *behaviors* (*R*^2^ = .249)			
*Predictors*	B	SE	β	*p*	B	SE	β	*p*	B	SE	β	*p*
Negative Emotionality	-0.026	.012	-.204	.029	-0.013	.017	-.068	.466	0.053	.014	.373	< .001
Extraversion	-0.042	.016	-.056	.566	0.040	.024	.162	.106	0.018	.019	.096	.352
Open-Mindedness	-0.012	.014	-.076	.409	0.059	.021	.263	.006	0.009	.017	.055	.571
Agreeableness	-0.014	.017	-.074	.417	-0.051	.025	-.188	.043	-0.050	.020	-.244	.012
Conscientiousness	0.110	.019	.542	< .001	0.097	.028	.328	< .001	0.023	.022	.103	.288

The third hypotheses suggested that VASSIP would display higher applicant reactions than BFI-2-S, especially regarding perception of organizational attractiveness. According with the mean differences displayed in Table 2, gamified assessment received higher scores only in three of the four applicant reactions: perception of comfort (*t* = -4.482, *p* < .001; Cohen’s *d* = -0.463), perceptions of predictive validity (*t* = -1.130, *p* = .026; Cohen’s *d* = -0.114), and perception of organizational attractiveness (*t* = -10.541, *p* < .001; Cohen’s *d* = -1.060). Thus, H3a was partially supported. Given that perception of attractiveness was higher for gamified assessment and that effect size was the highest of all comparisons, H3b was supported.

The last hypothesis proposed some associations between sociodemographic characteristics, Big Five, and applicant reactions. We developed predictive models of applicants’ reactions using gender, age, and the Big Five personality as predictors that are displayed in Table 5. Results showed that perception of fairness was determined by Negative Emotionality (β = -.257, *p* = .021), perception of comfort by age (β = -.340, *p* < .001), Negative Emotionality (β = -.266, *p* = .011), and Conscientiousness (β = .271, *p* = .012), and perception of predictive validity by Negative Emotionality (β = -.215, *p* = .050) and Conscientiousness (β = .240, *p* = .033). According with these results, we considered H4a as partially supported and H4b as not supported.

**Table 5:** Hierarchical regression analyses with applicant reactions as criteria.

	*Perception of fairness* (*R*^2^ = .116)				*Perception of comfort* (*R*^2^ = .221)				*Perception of predictive validity* (*R*^2^ = .137)				*Perception of attractiveness* (*R*^2^ = .112)			
*Predictors*	B	SE	β	*p*	B	SE	β	*P*	B	SE	β	*p*	B	SE	β	*p*
Gender	0.970	.573	.183	.094	0.015	.050	.003	.976	1.247	.848	.156	.150	0.396	.605	.071	.514
Age	-0.120	.066	-.191	.072	-0.201	.058	-.340	< .001	-0.101	.099	-.105	.312	-0.127	.070	-.192	.073
Negative Emotionality	-0.144	.061	-.257	.021	-0.139	.054	-.266	.011	-0.182	.092	-.215	.050	-0.119	.065	-.202	.069
Extraversion	-0.106	.084	-.143	.213	-0.009	.074	-.013	.903	-0.207	.127	-.184	.106	-0.151	.089	-.193	.095
Open-Mindedness	0.058	.072	-.086	.422	0.080	.064	.126	.214	0.135	.109	.131	.217	0.131	.077	.183	.091
Agreeableness	-0.014	.087	.017	.871	0.061	.076	.080	.427	0.030	.130	.024	.821	-0.094	.091	-.109	.308
Conscientiousness	0.125	.100	.140	.214	0.088	.088	.271	.012	0.325	.150	.240	.033	0.192	.106	.204	.072

*Note*. Reactions and personality scores refer to gamified assessment.

## Discussion

The study reported here is aimed to contribute to the investigation of gamified assessments as an alternative to personality questionnaires in personnel selection. In that sense, the present study is directed to contribute to three gaps in the literature: to advance the knowledge of the gamified assessments by analyzing one that use different game elements than the gamified assessments investigated in prior studies; to provide criterion-related validity, linking game scores with job performance; and to investigate which personal characteristics are related with higher applicant reactions to gamified assessments. Now we shall discuss all these results and put them into perspective.

Concerning the measurement of personality, our study found that reliability (Cronbach’s alpha) was slightly lower for the gamified assessment compared to the traditional assessment. From our point of view, this may be a signal of how gamification process may be detrimental for psychometric characteristics [8], at least until we know how to design better GRAs. Continuing with the results regarding personality, we also found that participants scored higher in Conscientiousness with VASSIP than with the original BFI-2-S. However, given that the items and response scale were the same, that participants answered both assessments in the same session, and that prior research on GRAs did not find similar results, we cannot discard that it was a methodological artifact. Other studies with gamified assessments have found similar results [12, 16, 22]. However, we believe that further research may help verify whether or not all gamified assessments share this characteristic.

The results of criterion-related validity showed that the regression models of task performance and counterproductive work behaviors were almost identical in traditional and gamified assessment, and results in contextual performance presented minor differences that may be due to the small sample size. However, from our point of view, these results are remarkable: until now, gamified assessments only have proven to be able to predict academic performance [13]. However, the present study extends to the main dimensions of job performance. Furthermore, although other GRAs such as gamefully designed assessments and game-based assessments have found support for task performance and contextual performance [14, 26], to the best of our knowledge this is the first study that investigates the use of gamified assessments (or any type of GRA) in regression models with counterproductive work behaviors as criterion. As mentioned, counterproductive work behaviors are a serious problem in modern organizations [45], so being able to identify using GRAs which characteristics of applicants’ are related to display these behaviors is always relevant.

This study also investigated applicants’ reactions. Aligned with previous research, our study found that gamified assessment obtained higher scores in all the reactions [12, 13, 18], but in the case of perception of fairness results are not statistically significant. The difference between traditional and gamified assessment with large effect size is organizational attractiveness, a consistent finding regardless of the GRA type [8]. We also stress that the effect size of the differences was small excepting for attractiveness. From a practitioner’s point of view, this means that gamified assessment may be advisable for job positions characterized by high competence among organizations as a way to increase the chances of the applicants wanting to work preferentially for one’s company than for others.

Continuing with applicant reactions, we found interesting associations between sociodemographic characteristics, personality, and applicants’ reactions. Gender, which shows positive associations with some reactions, is not included in the predictive models. This finding is common in research on applicant reactions [40], but it contradicts to previous results by Ellison and colleagues [19]. They suggested that the content of the game may be affecting applicant reactions, and if the game does include elements favored by a given gender, this result may disappear. It is true that there are considerable differences among their gamified assessment (i.e., a set of minigames to measure cognitive ability) and VASSIP, which is a story-driven measure of personality. Further research may help identify if the content of the game affects the applicant reactions of women and men differently. Regarding age, our results suggest that younger individuals, even in a sample with a limited range as ours, perceived more comfort with VASSIP. This result is aligned with the stereotype that young people have more positive feelings toward technology-based assessments. In terms of personality, our study indicates that low scores in Negative Emotionality were associated with more positive reactions regarding fairness, comfort, and validity, while high Conscientiousness was linked to more positive perceptions of comfort and validity. These findings are consistent with prior research with other assessment methods, although associations with other traits were also expected [46, 47]. It is also noteworthy that the perception of attractiveness was unrelated to sociodemographic characteristics and personality, suggesting that the higher positive reactions to GRAs compared to the traditional approach are due to the assessment method rather than the personal characteristics of the individuals being evaluated.

### Conclusions, limitations and further research

Gamified assessments are GRAs so close to the traditional assessments that they are expected to show similar psychometric characteristics to the measures on which they are based. Our results support this idea, showing that measuring personality with a gamified version of an existing scale (the short version of BFI-2-S) reaches very similar results to the original questionnaire. We also hypothesized that applicants’ reactions would be better for the gamified assessment than for the original scale, but this was only three of the four reactions analyzed. Personality and age determined applicant reactions, but not in the way we expected.

Considering all of these results, it seems that GRAs have the potential to be an alternative to personality questionnaires. Our findings with a gamified assessment indicate that even GRAs closely related to traditional assessments benefit from the gamification process. Consequently, developers of GRAs may choose to stay within their psychometric ’comfort zone’ to ensure measurement quality while achieving more favorable reactions that can capture candidates’ attention and leave them with positive impressions of the selection process. Regarding VASSIP, as it currently stands, it is not ready for use to make personnel assessment decisions. Further research is needed before its application in a real-world context, but may hold value for research purposes.

There is no study without limitations, and the present one is not an exception. The most relevant one is the sample size and its composition. Due to the small sample size, we are not sure whether some of the results can be generalized to the population, and some analyses cannot be carried out with reliably (e.g., Confirmatory Factor Analysis). Continuing with the sample, this study it was not conducted in an actual selection setting, or at least in a simulated one. Although this is typical in studies of new assessments that can be used for selection purposes, this is a limitation that should be addressed in the future. Research performed with real applicants would allow us to verify all these results about gamified assessments.

We also noted some limitations on procedure. First of all, counterbalancing the order in which the traditional *vs*. gamified assessment were presented would have been helpful to separate out score differences truly affected by the gamification vs. score differences associated with fatigue in taking the gamified version later into the two-hour test session. Additionally, we did not investigate the effect of the VASSIP story on participant behavior. Specifically, we did not examine whether the VASSIP story might encourage participants to behave in a certain way to achieve an ending they consider positive, such as scoring high in questions related to emotions to be considered ’human’ instead of an alien or a robot. We believe that this is an important issue for future research on VASSIP and other gamified assessments with a strong focus on narrative [e.g., 10, 13, 45]. Expanding on this idea, we want to encourage more research on game characteristics and their influence on the behavior of the person under evaluation. Besides that, the use of self-reports of job performance is also a limitation. We are aware that self-evaluations of performance tend to be higher than evaluations from peers or supervisors, but they also have advantages, namely that they are easy to collect and that people have the opportunity to observe all their own behaviors [41]. Besides that, the anonymity of the responses and the absence of consequences of the evaluation may contribute to honest responses. Another issue related with the measurement of job performance is that 22.4% of the sample lacks actual job experience. While these participants responded based on their behaviors as students, we acknowledge that this is a limitation. Considering all the aforementioned factors, further research on VASSIP and GRAs in general, should aim to explore measures of performance different from self-reports and consider collecting data from actual workers.

The low observed reliabilities are also a limitation of the present research. Although most of the values are acceptable for research purposes, a few measures have observed reliabilities that are suitable for actual personnel selection processes. Thus, the use of VASSIP in real practice is still not recommended, but we hope that further research will lead to its use in the future.

We also want to outline some ideas for further research. Firstly, given the differences between types of GRAs, it is further research comparing different types of GRA in the same study. Secondly, the study of faking of GRAs is still necessary. The first results are encouraging [11], but we need more evidence. Thirdly, we believe that GRA may be useful to assess constructs that are hard to measure with traditional assessments, such as dark personality traits. Game-based assessments, for example, allow observing behaviors instead of behavioral intentions or opinions, and in a more controlled environment than simulations. Finding evidence to support the measurement of constructs such as dark personality in this way could mean the difference between GRAs being just another assessment method or having salient features that make its use especially advisable in specific scenarios.

## Supporting information

S1 Checklist (DOCX)

S1 Appendix (RTF)
