# Supplementary material for: Are serious games an alternative to traditional personality questionnaires? Initial analysis of a gamified assessment
Source: PLoS One. 2024 May 2;19(5):e0302429. doi: 10.1371/journal.pone.0302429 (PMC11065274; doi:10.1371/journal.pone.0302429)
Supplement: S1 Checklist — (DOCX) [file pone.0302429.s001.docx]

**Human Participants Research Checklist**

***Complete the following if your study involved human participants or human participants’ data. These questions should be addressed for prospective and retrospective studies.***

1. Did you obtain ethics approval for this study?
   - If yes, please upload (file type “Other”) the original approval document you received from your ethics committee. If the original document is in another language, please also provide an English translation.

___ Uploaded _X_ N/A

- - If you did not obtain ethical approval, please explain why this was not required below.

The University of Zaragoza only have ethics committee for clinical studies and studies involving animals. Our study doesn't fit for any of these categories. Thus, we are not able to gather any ethical approval of our research. However, we follow the guidelines of the American Psychological Association (APA) Ethics Code (https://www.apa.org/ethics/code; sections 8. Research and 9. Assessment).

Participants were informed orally and by written about: (1) study purposes; (2) the type of information to be collected from them; (3) how data would be treated.; (4) that no personal information that allow identification (e.g., names, address, emails) was collected; (5) were ask for the research or its results.

The participants were informed about: (1) the purpose of the research; (2) their right to decline to participate or withdraw; (3) whom to contact for questions about the research or its results. The form of consent were obtained orally, because we are not gathering any data that allow identification of participants and the data were analysed anonymously.

1. If you prospectively recruited human participants for the study – for example, you conducted a clinical trial, distributed questionnaires, or obtained tissues, data or samples for the purposes of this study, please report in the Methods:
   1. the day, month and year of the **start and end** of the recruitment period for this study.
   2. whether participants provided informed consent, and if so, what type was obtained (for instance, written or verbal, and if verbal, how it was documented and witnessed). If your study included minors, state whether you obtained consent from parents or guardians. If the need for consent was waived by the ethics committee, please include this information.

_X__ Completed ___ N/A

1. If you are reporting a retrospective study of medical records or archived samples, please report in the Methods section:
2. the day, month and year when the data were accessed for research purposes
3. whether authors had access to information that could identify individual participants during or after data collection

___ Completed _X__ N/A
