# Supplementary material for: Are serious games an alternative to traditional personality questionnaires? Initial analysis of a gamified assessment
Source: PLoS One. 2024 May 2;19(5):e0302429. doi: 10.1371/journal.pone.0302429 (PMC11065274; doi:10.1371/journal.pone.0302429)
Supplement: S1 Appendix — (RTF) [file pone.0302429.s002.rtf]

Appendix
Perception of Comfort
I am satisfied with my results in the test/game.
I have been motivated during the test/game to achieve the best possible outcome.
It has been challenging for me to answer the questions/play the game. (Reversed)
I have felt comfortable with the test questions/while playing the game.
The test/game respects my privacy.


Perception of Predictive Validity
The test/game allows me to give false answers. (Reversed)
The test/game seems fair to me.
The test/game allows me to be evaluated objectively.
Candidates who perform well on the test/game will have better job performance.
The test/game is suitable for deciding who the best candidate is.
This test/game facilitates decision-making for recruiters.

Note. Items has been applied in Spanish in the present study. To access to the Spanish items, see https://doi.org/10.5093/jwop2023a18.
